# Can Team-Based Care Improve Patient Satisfaction? A Systematic Review of Randomized Controlled Trials

**DOI:** 10.1371/journal.pone.0100603

**Published:** 2014-07-11

**Authors:** Jin Wen, Kevin A. Schulman

**Affiliations:** 1 Department of Hospital Management and Health Policy, Institute of Hospital Management, West China Hospital, Sichuan University, Chengdu, China; 2 Duke Clinical Research Institute, Duke University School of Medicine, Durham, North Carolina, United States of America; 3 Department of Medicine, Duke University School of Medicine, Durham, North Carolina, United States of America; RAND Corporation, United States of America

## Abstract

**Background:**

Team-based approaches to patient care are a relatively recent innovation in health care delivery. The effectiveness of these approaches on patient outcomes has not been well documented. This paper reports a systematic review of the relationship between team-based care and patient satisfaction.

**Methods:**

We searched MEDLINE, EMBASE, Cochrane Library, CINAHL, and PSYCHOINFO for eligible studies dating from inception to October 8, 2012. Eligible studies reported (1) a randomized controlled trial, (2) interventions including both team-based care and non-team-based care (or usual care), and (3) outcomes including an assessment of patient satisfaction. Articles with different settings between intervention and control were excluded, as were trial protocols. The reference lists of retrieved papers were also evaluated for inclusion.

**Results:**

The literature search yielded 319 citations, of which 77 were screened for further full-text evaluation. Of these, 27 articles were included in the systematic review. The 26 trials with a total of 15,526 participants were included in this systematic review. The pooling result of dichotomous data (number of studies: 10) showed that team-based care had a positive effect on patient satisfaction compared with usual care (odds ratio, 2.09; 95% confidence interval, 1.54 to 2.84); however, combined continuous data (number of studies: 7) demonstrated that there was no significant difference in patient satisfaction between team-based care and usual care (standardized mean difference, −0.02; 95% confidence interval, −0.40 to 0.36).

**Conclusions:**

Some evidence showed that team-based care is better than usual care in improving patient satisfaction. However, considering the pooling result of continuous data, along with the suboptimal quality of included trials, further large-scale and high-quality randomized controlled trials comparing team-based care and usual care are needed.

## Introduction

Team-based care has been offered as an improvement in care delivery, especially for the treatment of patients with complicated medical conditions. When properly implemented, team-based approaches have been shown to improve clinical decision making [1], [2]. In practice, team-based care takes many forms, such as inpatient care management teams or multidisciplinary disease-oriented care programs. Teams may be large or small and are found in a variety of practice settings, from private clinics to academic medical centers [3]–[5]. Given this variation, it is therefore difficult to define team-based care. Mitchell et al [6] defined team-based care as “the provision of health services to individuals, families, and/or their communities by at least 2 health providers who work collaboratively with patients and their caregivers—to the extent preferred by each patient—to accomplish shared goals within and across settings to achieve coordinated, high-quality care.”

Team-based care has been reported as an important attribute of patient-centered care [7]. In particular, 1 practice model that has been promoted as a way to improve patient care delivery is the patient-centered medical home (PCMH). The Agency for Healthcare Research and Quality defined the PCMH by five attributes: (1) a patient-centered orientation, (2) comprehensive, team-based care, (3) coordinated care, (4) access to care, and (5) a systems-based approach to quality and safety [8].

Additionally, patient satisfaction has become an increasingly important and commonly used indicator for measuring the quality of health care. Patient satisfaction has also become a proxy, and an effective indicator, to measure the success of doctors and hospitals. In the United States, physician bonuses are linked to patient evaluations of a doctor's personal interaction with them. In the United Kingdom, general practitioner contracts have been implemented, which provide bonuses of up to 30% of a general practitioner's income for reaching quality targets [9]. The point system offers rewards not only for clinical performance measures of quality, but also for conducting patient surveys and acting on patient feedback to improve care. These developments highlight how higher patient satisfaction leads to benefits for the health industry in a number of ways, which have been documented by various studies [10], [11].

One of the main purposes of team-based health care interventions is improving the patient experience [12]. However, some uncertainty remains about the relationship between team-based care and patient satisfaction [13]–[15]. Moreover, to our knowledge, there has not been a systematic review exploring the link between the team approach to patient care and patient satisfaction. Considering the popularity of team-based interventions and the importance of the patient experience, we sought to systematically review the relationship between these 2 factors.

## Methods

### Search Strategies

The institutional review board of the Duke University Health System approved the study. We conducted systematic literature searches using MEDLINE, EMBASE, the Cochrane Library, CINAHL, and PSYCHOINFO. We searched all databases from inception to October 8, 2012. We also evaluated the reference lists of retrieved papers for potential inclusion of additional articles.

In each database, we searched for English-language articles that included the following 3 concepts: (1) patient satisfaction, (2) team-based care, and (3) a randomized controlled trial design. In MEDLINE, we use Medical Subject Headings (MeSH) for all search terms unless otherwise noted, and we searched for the terms related to the 3 concepts of interest (**Appendix S1**). For patient satisfaction, we used the search terms “patient satisfaction” and “consumer satisfaction.” For team-based care, we used the search terms “patient care team,” “nursing team,” “teamwork,” “team work,” “multidisciplinary team,” “interdisciplinary team,” “shared care,” “collaborative care,” and “integrated care.” For randomized controlled trial design, we applied the Scottish Intercollegiate Guidelines Network search filter [16]. In the remaining databases, we defined the search terms as keywords only.

### Study Selection

We reviewed the abstracts of all citations and retrieved studies based on the following inclusion criteria: (1) the study was designed as a randomized controlled trial; (2) interventions included team-based care and non-team-based care (or usual care); and (3) outcomes included an assessment of patient satisfaction. We defined team-based care as a provision of health services by at least 2 disciplines and 2 health providers who work collaboratively with shared goals. Because the description of instructions and information provided in usual care was often insufficient [17], we defined usual care as: (1) an intervention by a provider alone, or (2) “usual care,” “routine care,” “standard care,” “conventional care,” or similar terms as a control group mentioned in randomized controlled trials. We excluded articles and trial protocols with different settings between intervention and control.

### Data Extraction and Quality Assessment

We considered trials for inclusion, assessed the quality of eligible studies, and extracted data using a standardized protocol and reporting form. Two reviewers independently reviewed each article for eligibility. For each study, 1 reviewer extracted the data and assessed the risk of bias while a second reviewer verified the accuracy. Disagreements were resolved by consensus. On the basis of prior work on patient satisfaction and a literature review, we chose 1 overall item (eg, “How do you rate the hospital overall?” or “How do you rate your overall satisfaction?”) to assess patients' overall satisfaction with their hospital experience. If no “overall satisfaction” assessment was provided, we took the item “satisfaction with the care or similar” description as the overall satisfaction.

We assessed the quality of each trial according to the Cochrane Handbook for Systematic Reviews of Interventions [18]. We used the following quality assessment items: random sequence generation, allocation concealment, blinding of participants and personnel, blinding of outcome assessors, incomplete outcome data, and other sources of bias. We rated each item on a 1-to-3 scale, where 1 represented a low risk of bias, 2 a high risk of bias, and 3 an unclear risk of bias.

### Data Analysis

We express dichotomous data as odds ratios (OR) with 95% confidence intervals (CIs). We express continuous data as standardized mean differences (SMDs) with 95% CIs. We tested statistical heterogeneity using the *I^2^* test. If results showed substantial heterogeneity (*I* ^2^>60%), we conducted a subgroup or random-effects analysis to examine potential sources of heterogeneity (eg, length of follow-up, trial quality, and intervention variation). Results were considered statistically significant at 2-sided *P*<.05. We used RevMan 5.1 (Cochrane Collaboration) for all analyses.

## Results

**Figure 1:**
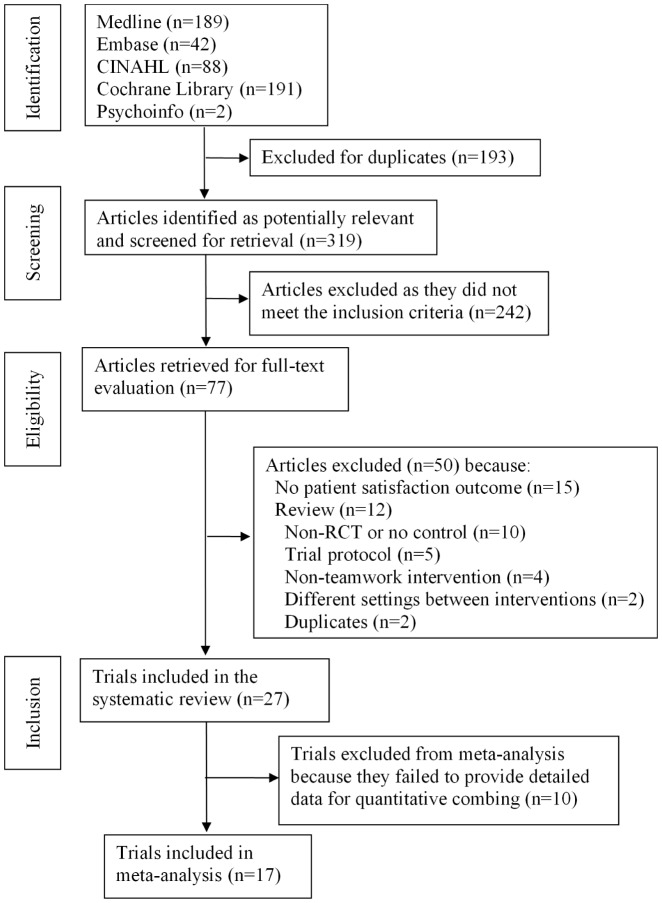
Flow Diagram of Trial Selection Process for the Systematic Review.

Our literature search yielded 319 citations, of which 77 were screened for further full-text evaluation. Of those, we included 27 citations [13]–[15], [19]–[42] this systematic review (**Table 1**). **Figure 1** shows an overview of the study selection process. Of the 27 included papers, 2 [27], [35] reported on the same trial, but the outcome data were from different follow-up periods. **Table 2** shows that trial quality varied among the 26 trials. Twelve reported sequence generation, 6 described allocation concealment, 2 clearly adopted blinding of participants and personnel, 9 reported blinding of outcome assessors, 8 reported a low risk of bias on incomplete outcome data, and 4 were judged to have low risk of other sources of bias.

**Table 1:** Characteristics of Included Studies.

Study	Patient Characteristics	Setting	Study Length	Intervention
Zimmer 1985	Older patients with chronic or terminal illness	Home	6 mo	Home health care team vs control group
Williams 1987	Frail patients ≥65 years	PHC	12 mo	GACS vs traditional group
Hughes 1992	Patients with terminal illness	Home	6 mo	Hospital-based home care vs customary care
Turnbull 1996	Pregnant women	Hospital	7 mo	Shared care vs midwife managed care
Ronald 1996	Patients ≥55 years with multiple chronic illnesses	PHC	8 mo	GEM vs UPC
Beck 1997	Patients ≥65 years with chronic illness	PHC	12 mo	Cooperative health care clinic vs usual care
Coleman 1999	Frail patients ≥65 years	PHC	24 mo	Chronic care clinic vs usual care
Sadur 1999	Patients 16 to 75 years with diabetes	PHC	6 mo	DCCC vs usual care
Rost 2001	Adults with depression	PHC	6 mo	QuEST vs usual care
Tijhuis 2002	Patients with rheumatoid arthritis	PHC	13 mo	Inpatient team care vs clinical nurse specialist
Unutzer 2002, Hunkeler 2006^d^	Patients ≥60 years with depression	PHC	12 mo	IMPACT vs usual care
Litaker 2003	Patients with hypertension and diabetes	Hospital	12 mo	Nurse practitioner-physician team vs physician only usual care
Scott 2004	Patients ≥60 years with chronic illness	PHC	24 mo	Cooperative health care clinic vs control group
Byng 2004	Patients with mental illness	PHC	18 mo	Mental health link intervention vs usual service provision
Smith 2004	Adults with diabetes	PHC	18	Diabetes shared care model vs usual care
Preen 2005	Inpatients with chronic cardiorespiratory illness	Hospital	0.25 mo	Care plan group vs control group
Johnson 2005	Patients 18 to 65 years with acute mental illness	Community	2 mo	Crisis resolution team vs standard care
Scott 2005	Patients with gastrostomy	Hospital and home	12 mo	Nutrition support team vs standard practice group
Garety 2006	Patients 16 to 40 years with early psychosis	South London and NHS Trust	18 mo	Lambeth early onset team care vs standard care
Garcia-Aymerich 2007	Patients with chronic obstructive pulmonary disease	Hospital	12 mo	Integrated care vs conventional care
Brumley 2007	Patients with terminal illness	HMO	4 mo	IHPC vs usual care
Gade 2008	Adults with life-limiting illness	Hospital	6 mo	IPCS vs usual care
Hunt 2008	Patients with uncontrolled hypertension	PHC	12 mo	Phycisian pharmacist collaborative care vs usual care
Pape 2011	Adults with diabetes	PHC	24 mo	Physician-pharmacist team-based care vs control arm
Fihn 2011	Patients with stable ischemic heart disease	PHC	12 mo	Collaborative care vs usual care
Ell 2011	Adults with cancer	PHC	24 mo	ADAPt-C collaborative care vs enhanced usual care

Abbreviations: DCCC, diabetes cooperative care clinic; GACS, geriatric ambulatory consultive service; GEM, geriatric evaluation and management; HMO, health maintenance organization; IHPC, in-home palliative care; IMPACT, improving mood-promoting access to collaborative treatment; IPCS, interdisciplinary palliative care service; PHC, primary health center or clinic; QuEST, quality enhancement by strategic teaming; UPC, usual primary care.

**Table 2:** Quality Assessment of Included Studies.

Study	Random Sequence Generation	Allocation Concealment	Blinding of Participant and Personnel	Blinding of Outcome Assessors	Incomplete Outcome Data^a^	Other Bias^b^
Zimmer 1985	unclear	high risk	high risk	high risk	high risk	unclear
Williams 1987	unclear	low risk	low risk	low risk	low risk	unclear
Hughes 1992	unclear	unclear	unclear	unclear	unclear	unclear
Turnbull 1996	unclear	low risk	high risk	high risk	low risk	unclear
Ronald 1996	unclear	unclear	high risk	high risk	low risk	unclear
Beck 1997	low risk	unclear	unclear	unclear	high risk	unclear
Coleman 1999	unclear	unclear	unclear	unclear	high risk	high risk
Sadur 1999	low risk	unclear	unclear	unclear	high risk	unclear
Rost 2001	unclear	unclear	unclear	low risk	high risk	high risk
Tijhuis 2002	low risk	low risk	high risk	low risk	unclear	low risk
Unutzer 2002, Hunkeler 2006^c^	low risk	low risk	unclear	low risk	low risk	low risk
Litaker 2003	unclear	unclear	unclear	unclear	low risk	unclear
Scott 2004	low risk	unclear	high risk	high risk	high risk	unclear
Byng 2004	unclear	unclear	low risk	low risk	high risk	high risk
Smith 2004	low risk	unclear	unclear	unclear	low risk	high risk
Preen 2005	unclear	unclear	unclear	unclear	high risk	high risk
Johnson 2005	unclear	low risk	high risk	high risk	low risk	unclear
Scott 2005	unclear	unclear	high risk	unclear	low risk	low risk
Garety 2006	low risk	low risk	high risk	low risk	high risk	low risk
Garcia-Aymerich 2007	low risk	unclear	unclear	low risk	high risk	unclear
Brumley 2007	low risk	unclear	unclear	low risk	high risk	unclear
Gade 2008	low risk	unclear	unclear	unclear	unclear	high risk
Hunt 2008	low risk	unclear	high risk	low risk	high risk	unclear
Pape 2011	unclear	unclear	high risk	unclear	high risk	unclear
Fihn 2011	unclear	unclear	unclear	unclear	unclear	high risk
Ell 2011	low risk	unclear	unclear	unclear	high risk	high risk

a Dropout rate less than 15% denotes low risk of bias.

b Other sources of bias that are relevant only in certain circumstances such as particular trial design (eg, recruitment bias in cluster-randomized trials) and bias due to early stopping.

c Unutzer 2002 and Hunkeler 2006 are the same trial, but reported different follow-up outcome data.

A total of 15,526 participants are represented in the 26 trials. Of these, 6768 participants (43.6%) were assigned to team-based care and 8758 (56.4%) to usual care. All but 9 of the trials conducted statistical analyses based on the intention-to-treat principle. **Table 1** summarizes the main characteristics of each trial. Study length varied from 1 week to 24 months. The overall median follow-up period was 12 months. Thirteen trials focused on frail older adults or patients with chronic disease, 5 enrolled patients with mental health conditions, 5 studied terminal illness or patients with cancer, and the remaining 3 enrolled other patients.

### Structure and Process of Team-Based Care and Usual Care

**Table 3** shows the characteristics of the structure and process of the interventions. Fourteen articles reported the number of team members for team-based care, with a median of 4 (range, 2–12). Five articles reported the number of team members for usual care, with a median of 1 (range, 1–2). Nine papers reported that team members for team-based care had credentials, and 3 papers reported that members exercising usual care were certified. Similar proportions were found in task deployment between the interventions (25 for team-based care, 8 for usual care). Although there was no information on response time for usual care, 8 papers reported the response time for team-based care, with a median response time of 120 minutes (range, 90–540).

**Table 3:** Structure and Process Summary for Team-Based Care and Usual Care.

Item	Team-Based Care	Usual Care
Structure		
Number of team members, median (range)	4 (2–12)^a^	1 (1–2)^b^
Credential, No. (%)		
Yes	9 (33.3)	3 (11.1)
Not applicable	18 (66.7)	24 (88.9)
Deployment, No. (%)		
Yes	25 (92.6)	8 (29.6)
Not applicable	2 (7.4)	19 (70.4)
Response time, median (range), min	120 (90–540)^c^	—d
Process		
Care protocol, No. (%)		
Yes	24 (88.9)	2 (7.4)
Not applicable	3 (11.1)	25 (92.6)
Training, No. (%)		
Yes	10 (37.0)	2 (7.4)
Not applicable	17 (63.0)	25 (92.6)
Medication administration, No. (%)		
Yes	14 (51.9)	1 (3.7)
Not applicable	13 (48.1)	26 (96.3)
Regular meetings, No. (%)		
Yes	16 (59.3)	2 (7.4)
Not applicable	11 (40.7)	25 (92.6)
Interdependent, No. (%)		
Yes	16 (59.3)	1 (3.7)
Not applicable	11 (40.7)	26 (96.3)
Shared decision, No. (%)		
Yes	10 (37.0)	1 (3.7)
Not applicable	17 (63.0)	26 (96.3)

a Fourteen of 27 papers reported the number of team members for team-based care.

b Five of 27 papers reported the number of team members for usual care.

c Eight of 27 papers reported the response time for team-based care.

d No papers reported the response time for usual care.

Twenty-four articles reported that there was a care protocol for team-based care, and 2 articles reported that there was a care protocol for usual care. Ten papers reported that those conducting team-based care were trained before issuing care, and 2 papers reported that those conducting usual care were trained before issuing care. Fourteen papers reported that the team-based approach included administering medications, and 1 reported that usual care included medications. Sixteen articles reported that team-based care included regular team meetings to review care, whereas 2 reported that usual care included regular meetings. Sixteen articles reported that members of team-based care were interdependent, whereas 1 reported interdependence in usual care. In addition, 10 articles reported that team-based care adopted a “shared decision” model, while 1 stated this approach was used in usual care.

### Overview of Patient Satisfaction

Six articles (22.2%) reported that patient satisfaction was a primary outcome, 2 (7.4%) described satisfaction as a secondary outcome, and the remaining 19 (70.4%) did not define the order of patient satisfaction as an outcome measure. Eighteen papers (66.7%) described the satisfaction measurement tool items, of which 5 used 1 item to measure patient satisfaction. The maximum number of items was 35, and the median number of items was 8. Of the 18 papers that described satisfaction measurement tools, 13 clearly stated that the satisfaction assessment measure had been validated prior to use in the study.

### Effect Sizes of Patient Satisfaction: Dichotomous Data

**Figure 2:**
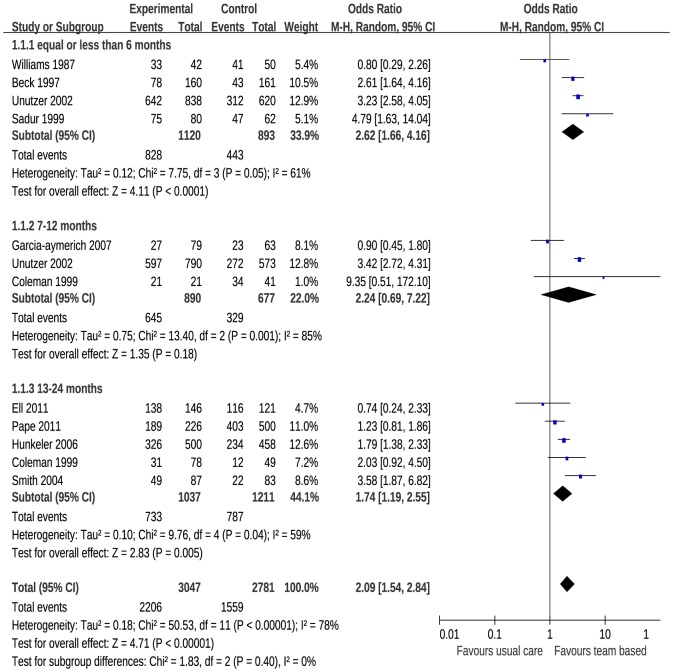
Meta-Analysis of Binary Data on the Effect of Team-Based Care on Patient Satisfaction.

Thirteen studies reported dichotomous data of patient satisfaction, of which 7 reported no statistical difference (*P*>.05) in patient satisfaction between team-based care and usual care. The remaining 6 papers showed that patients who received team-based care reported higher satisfaction than those treated by usual care. However, 3 papers did not provide primary data and were therefore excluded from the analysis. The test of overall heterogeneity for the 10 included trials resulted in *I* ^2^ = 78% and *P*<0.001 **Figure 2** shows that team-based care had a positive effect on patient satisfaction compared with usual care for papers which measured patient satisfaction as a dichotomous outcome (OR, 2.09; 95% CI, 1.54 to 2.84).

### Effect Sizes of Patient Satisfaction: Continuous Data

**Figure 3:**
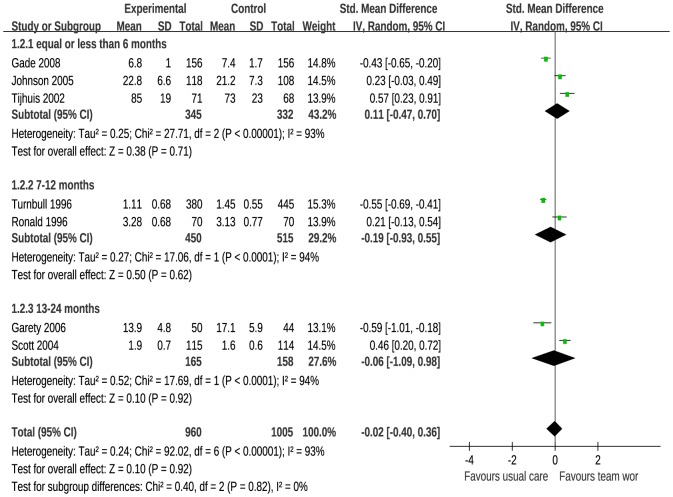
Meta-Analysis of Continuous Data on the Effect of Team-Based Care on Patient Satisfaction.

Fourteen studies reported continuous data for patient satisfaction, of which 7 reported no statistical difference (*P*>.05) in patient satisfaction between team-based care and usual care. The remaining 7 papers reported statistically significant differences between the 2 interventions. Seven papers did not provide means and/or standard deviations and thus were excluded from the analysis. The test of overall heterogeneity for the 7 included trials resulted in *I* ^2^ = 93% and *P*<0.001. **Figure 3** shows that there was no significant difference in patient satisfaction between team-based care and usual care for papers reporting outcomes using a continuous measure (SMD, −0.02; 95% CI, −0.40 to 0.36).

## Discussion

Team-based care is a growing trend in care delivery intended to have significant benefits for patients ranging from more informed decision making for complex conditions to improved access and reduced cost of care. Although these benefits have been described for team-based care, evidence for the impact of this approach on patient satisfaction remains underdeveloped. In this analysis, we found inconsistent results on the effectiveness of team-based care on patient satisfaction. In studies that reported patient satisfaction as a dichotomous outcome, we found a positive result for the relationship between team-based care and patient satisfaction [15], [20], [23], [24], [27], [31], [35], [37], [40], [42]. Yet, for studies that reported patient satisfaction as a continuous outcome, we found no relationship between team-based care and patient satisfaction [13], [14], [22], [26], [29], [34], [38].

Our findings reveal that trial quality is suboptimal in this literature. Most included trials failed to report random sequence generation, allocation concealment, and blinding. Many trials also had a high or unclear risk of bias, reported incomplete outcome data, and had other sources of bias. Similar results have been reported by other studies [43], [44]. Furthermore, more than one-third of eligible trials did not provide complete primary data, such as mean and standard deviation for the patient satisfaction outcome measure, which led to their exclusion from our analysis.

Teamwork is thought to be a prerequisite for good practice in health care. However, teams are diverse and range in a variety of factors, including number of members and disciplines. Therefore, it is necessary to clearly report the structure and process of team-based care and to explicitly describe the structure and process of usual care for comparison. These details are necessary for understanding the team-based concept being assessed, assessing the effectiveness of team performance, and understanding structural and procedural factors that may also affect the level of performance for comparative usual care. Unfortunately, many trials did not explicitly describe the care structure and process for team-based approaches, and most failed to do so for usual care. Generally, trial authors preferred depicting the structure and process of team-based care while omitting those of usual care. Measures of the process of care delivery for both intervention and comparison groups are required to adequately assess the effect of team-based care on clinical outcomes.

Patient satisfaction is increasingly the focus of research and evaluation of health care interventions and is identified as an important quality outcome indicator of health care in the hospital setting [45], [46]. There are a number of methods available for assessing patient satisfaction. While multi-item questionnaires provide detail for rigorous studies, single-item measures offer simplicity and speed for the purposes of clinical audits. Whatever the method of assessment, authors should consider the performance of the assessment instrument in the design of their study. Over half of the trials included in this study failed to report the validation status of the measure used to assess patient satisfaction.

The purpose of teamwork is to improve communication and partnership among health providers and patients [47], promote quality and safety, and enhance patient satisfaction. While the merits of teamwork are well documented and the teamwork model is widely used, the positive relationship between teamwork and health care outcomes, particularly patient satisfaction, is not well documented. Our review provided some evidence to support the positive link between team-based care and patient satisfaction; however, this result is not consistent when assessing the literature based on how the outcome data were reported.

Our review has some limitations. First, there is no standard definition of a team. Studies evaluating collaborations of specific personnel might not have described their model as a “team” and therefore would not be included in our study. Second, team-based care was not a consistent construct across the trials we reviewed. Moreover, measures of patient satisfaction as an outcome also varied significantly, as did the methods of reporting the outcome measures in the primary data. To address these concerns, we reported relative effect size indicators (ORs and SDMs) and performed a random effects meta-analysis. Third, the quality of trials was suboptimal. The results we report are inconsistent between studies that reported a dichotomous outcome measure and those that reported a continuous measure. We were unable to conduct a subgroup analysis of study quality due to the small number of studies in both subgroups. Finally, 10 articles did not report the necessary primary data to be included in the analysis and were excluded from this review. Although we attempted to contact the authors of these trials, we were unsuccessful in acquiring the necessary data for inclusion or lacked sufficient contact information.

In conclusion, our meta-analysis shows there is some evidence that team-based care might be better than usual care in achieving patient satisfaction. Nevertheless, considering the pooling result of continuous data, along with the suboptimal quality of included trials, further large-scale and high-quality randomized controlled trials comparing team-based care versus usual care, combined with clear definitions of usual and team-based care, are needed.

## Supporting Information

Appendix S1 **Search Strategy for MEDLINE.**
(DOCX)

Checklist S1 **PRISMA Checklist.**
(DOC)
