# Supplementary material for: Can Team-Based Care Improve Patient Satisfaction? A Systematic Review of Randomized Controlled Trials
Source: PLoS One. 2014 Jul 11;9(7):e100603. doi: 10.1371/journal.pone.0100603 (PMC4094385; doi:10.1371/journal.pone.0100603)
Supplement: Appendix S1 — Search Strategy for MEDLINE. (DOCX) [file pone.0100603.s001.docx]

**Appendix**. Search Strategy for MEDLINE

| 1 | Randomized Controlled Trials as Topic/ |
| --- | --- |
| 2 | randomized controlled trial/ |
| 3 | Random Allocation/ |
| 4 | Double Blind Method/ |
| 5 | Single Blind Method/ |
| 6 | clinical trial/ |
| 7 | clinical trial, phase i.pt. |
| 8 | clinical trial, phase ii.pt. |
| 9 | clinical trial, phase iii.pt. |
| 10 | clinical trial, phase iv.pt. |
| 11 | controlled clinical trial.pt. |
| 12 | randomized controlled trial.pt. |
| 13 | multicenter study.pt. |
| 14 | clinical trial.pt. |
| 15 | exp Clinical Trials as topic/ |
| 16 | or/1-15 |
| 17 | (clinical adj trial$).tw. |
| 18 | ((singl$ or doubl$ or treb$ or tripl$) adj (blind$3 or mask$3)).tw. |
| 19 | PLACEBOS/ |
| 20 | placebo$.tw. |
| 21 | randomly allocated.tw. |
| 22 | (allocated adj2 random$).tw. |
| 23 | or/17-22 |
| 24 | 16 or 23 |
| 25 | case report.tw. |
| 26 | letter/ |
| 27 | historical article/ |
| 28 | or/25-27 |
| 29 | 24 not 28 |
| 30 | Patient Care Team/ |
| 31 | Nursing, Team/ |
| 32 | (teamwork or "team work").mp. [mp=title, abstract, original title, name of substance word, subject heading word, protocol supplementary concept, rare disease supplementary concept, unique identifier] |
| 33 | Multidisciplinary team?.tw. |
| 34 | Multi disciplinary team?.tw. |
| 35 | Interdisciplinary team?.tw. |
| 36 | 30 or 31 or 32 or 33 or 34 or 35 |
| 37 | Patient Satisfaction/ |
| 38 | Consumer Satisfaction/ |
| 39 | 37 or 38 |
| 40 | 29 and 36 and 39 |
